# The Coming Coronavirus Crisis: What Can We Learn?

**DOI:** 10.1007/s10272-020-0878-0

**Published:** 2020-04-01

**Authors:** Matteo Lucchese, Mario Pianta

**Affiliations:** Faculty of Political and Social Sciences, Scuola Normale Superiore, Palazzo Strozzi, Piazza Strozzi, 50123 Firenze, Italy

## Abstract

The coronavirus pandemic is bringing with it the prospect of severe financial and economic crises. The article investigates its economic consequences in terms of financial instability, economic recession, lower incomes and policy challenges at the national and European levels. What are some of the lessons that can be learned? This article argues that health is a global public good. Public health and welfare systems are crucial alternatives to the market and universal public health is a key element of an egalitarian policy.

The coronavirus pandemic is a major health emergency.[1] As of 27 March, the World Health Organization (WHO) reported over 500,000 cases and more than 24,000 deaths worldwide. In China, where the virus started, the infection seems to have stopped after reaching 81,000 cases; in Italy – the second most affected country with over 80,000 confirmed cases on 26 March – the pandemic has not yet slowed. In many of the other 196 territories infected, the virus is spreading at a sustained pace and several countries, including the United States, are introducing drastic measures to address the spread of the virus.

The pandemic is bringing with it a major economic and financial crisis. Facing the economic consequences of coronavirus is a major challenge for national governments, European institutions and the international system. There is an urgent need to understand the extent of the crisis and the nature of the health, social and economic problems we face as well as to update the policies that have led to this crisis.

## The financial crisis has arrived

With fears of a harsh credit crisis and a major collapse in economic activity, the spreading of the pandemic crashed financial markets all over the world. Between 19 February and 20 March 2020, the S&P500 index at the Wall Street Stock Exchange lost 32%. In London, the fall of the FTSE100 index was in the same range. In Italy – the first European country to be infested by the pandemic – the Milan FTSE MIB index lost 38%.

This is close to a financial crash. Until now, there has not been a specific factor that could replicate the role that the collapse of 'subprime' mortgages and the bankruptcy of Lehman Brothers played in 2007 and 2008 leading to a 50% fall of the US Stock Exchange. This time, the slowdown of the economy could simply make the mass of private debt unpayable; according to the OECD, world private corporations have an outstanding debt of 13.5 trillion US dollars (Goodman, 2020), after a decade of huge debt expansion, made easier by interest rates close to zero. Or the crisis could erupt in some unexpected point – for instance, the dysfunctionality of the US health system, a bankruptcy of US private health insurance companies or a new public debt crisis in Europe (Kerry, 2020).

In response to the crisis, the US Federal Reserve has announced drastic measures, including an extensive programme of quantitative easing and several emergency actions to support the flow of credit to consumers, businesses and municipalities. The European Central Bank (ECB) unveiled a 750 billion euro emergency bond plan to strengthen banks' capital and support companies' li-

* The views expressed do not necessarily reflect those of the institutions with which the authors are affiliated.

1 For an earlier article on the economic consequences of the pandemic, see Lucchese and Pianta (2020).

**Matteo Lucchese**, Istat, Rome; and Scuola Normale Superiore, Florence, Italy.

**Mario Pianta**, Scuola Normale Superiore, Florence, Italy.

quidity demand. The ECB is ready to offer over 1.1 trillion euro to the financial markets. “There are no limits to our commitment to the euro. We are determined to use the full potential of our tools, within our mandate”, ECB President Christine Lagarde said (ECB, 2020).

### The United States

The epicentre of the financial crisis is likely to be in the United States. In February 2020, Wall Street stock indices were double their 2007 values, a level which is barely justified by the conditions of the real economy. Extreme financial speculation has been fuelled above all by the belief that, with Donald Trump in the White House, policies to support finance and business and tax breaks for the rich would allow Wall Street to continue its expansion. The recent expansionary measures taken by the US Federal Reserve in order to stabilise the financial markets, shocked by the spreading of the virus and the drop in oil prices, were still going in that direction.

Before the coronavirus pandemic, the most likely scenario for the US was a continuation of financial expansion – artificially supported by fiscal and monetary policies – until the presidential elections in November 2020. Typically, there is never a recession on the eve of a US presidential election; adjustments and crises usually occur the following year. If that were the case, Trump would likely win again, riding on the wave of a growing economy with low unemployment, and relying on the right-oriented radicalisation of his core electorate.

Now the scenario has completely changed. The ability of the United States to control the pandemic is difficult to assess; government actions are now catching up after the early denial of the severity of coronavirus infections. Pressed by the spreading of the pandemic and a risk of a severe recession in the US, the Trump administration has planned to launch with dangerous delays and strong divisions in Congress – a nearly 2 trillion dollar plan to shore up the economy. It includes supporting the costs of an underfunded and highly unequal healthcare system, helping firms, extending unemployment benefits and family leave, and even sending money directly to US citizens. With a 2019 US GDP of 21.2 trillion dollars, the US emergency plan amounts to about 9.5% of GDP. Still, Trump may prove to be an inadequate leader in the face of this emergency (Krugman, 2020). There is a possibility that everything might collapse: starting with a severe drop in US and world economic growth, a financial crash, Trump losing the election and the likely Democratic candidate former Vice President Joe Biden being left to restore some order in 2021. In terms of world order, a continuation of ‘systemic chaos’ could be expected, with the further decline of US leadership and the rising influence of Asia and China.[2]

### Europe

Economies on this side of the Atlantic are facing severe difficulties – many of them of their own making. The ECB’s new plan to overcome the crisis came after days of a lack of clarity and divisions within the board. On 12 March, the first ECB decision to provide new liquidity was accompanied by a disastrous statement by Lagarde: “[W]e are not here to close spreads”, taken from a phrase by a German Member of the Executive Board of the ECB (Lagarde and de Guindos, 2020). A major worsening of the spread in interest rates between Italian and German government bonds and a stock market fall immediately followed the statement. The President of the Italian Republic Sergio Mattarella, with an unprecedented intervention, immediately replied that “initiatives of solidarity and not moves that can hinder Italy’s actions” (2020) are expected from Europe, leading to a slight correction of Lagarde’s view: “I am fully committed to avoid any fragmentation in a difficult moment for the euro area” (Lagarde and de Guindos, 2020).

A few days later, the Board of Directors decided to launch a massive injection of liquidity into the economic system. However, this unprecedented clash between Italy and the European Central Bank reveals the deep divisions in the ECB governing council and how far German and French strategies are from the interests of Italy and Southern Europe. European institutions appear unable to handle an economic emergency. Without a radical change, the ‘fragmentation’ of the euro area could become one of the effects of the pandemic.

## The coming economic recession

The coronavirus pandemic is bringing on a major economic crisis. In the last OECD Economic Outlook (OECD, 2020), annual global GDP growth was projected to fall to 2.4% in 2020, from an already weak 2.9% in 2019, with a possible contraction of GDP in the first quarter of 2020. GDP growth in China could be below 5% this year, with a marked downward correction. In fact, there is evidence that things will get worse. The most recent data on China show that the industrial production index fell by 13.5% in the first two months of 2020, the most dramatic fall since the early 1990s. The spread of the pandemic in Europe and the US could make the fall in GDP much larger, with stagnation or recession in all of Europe, and a significant

2 On the instability of the world system, see Arrighi (1994) and Arrighi and Silver (1999).

fall – maybe in the range of 5% – for the most fragile economies of Southern Europe.

The drivers of the crisis are the stoppages in production and consumption in the months of most acute diffusion of the pandemic (the first quarter in China and in Italy, the months from March onwards in the rest of Europe and in the US). Whole sectors, such as air travel, transportation, tourism and restaurants, have completely stopped. As European economies are closely integrated in global value chains, they will probably suffer from a 'supply-chain contagion' (Baldwin and Weder di Mauro, 2020). Major negative economic effects are associated with the loss of employment and wages, which can only be compensated to a limited extent by the much-needed income support measures introduced by governments (guaranteed incomes, tax relief, etc.). The resulting fall in demand will further slow down production, while the increase in health expenditure is unlikely to have significant expansionary effects on the economy as a whole.

Data on Italy may provide some indication of what could happen in Europe as a whole. According to Confindustria, the main Italian business association, 20% of companies have experienced strong negative effects; some sectors, such as tourism, will be affected far beyond the most acute moments of the pandemic. Furthermore, income support measures may not translate into increased domestic production, but may lead to greater imports (as it has happened in the case of face masks and respiratory machinery). After the 2008 crisis, Italy and Southern Europe experienced a 20% fall in industrial production that later became permanent. A similar, further weakening of the economies of the European periphery could result from the coming crisis.

Traditional economic policy tools could be ineffective in combating the consequences of the coronavirus. European monetary policy will not make a big impact on the real economy. The indirect stimulus of expansive fiscal policies or tax relief could have a modest impact in the short term. The most effective tool for containing the crisis could be a large increase in public spending for the provision of public services, the purchase of domestically produced goods and investment in new production activities in the context of a green industrial policy.

## The coming crisis of Europe

The 2008 crash and the succeeding European debt crisis in 2011 have made apparent the inadequacy of European institutions and policies. These crises have turned into a decade of recession and stagnation for Southern European countries. A major legitimation crisis has infested the European Union, contributing to the eventual exit of the UK from the Union. This is a scenario that could happen again, as Europe lacks the ability to quickly intervene and address the economic consequences of the coronavirus pandemic.

In the unfolding of the coronavirus crisis, the European Commission (EC) decided to temporarily weaken two of the most criticised pillars of European economic policies in the past 30 years. First, the EC launched a temporary revision of state aid measures to ensure support for workers' incomes and the necessary liquidity for businesses – even rescue them with partial nationalisation if necessary; then the EC activated the 'general escape clause', which temporarily suspended the Stability and Growth Pact, providing governments with greater fiscal space to "pump as much money as it takes into the system" (EC, 2020). This radical change in fiscal policy has allowed the launch of massive national plans to fight the coronavirus epidemic. Germany has already announced a fiscal expansion of over 150 billion euro, 4.3% of GDP, overcoming the maximum debt limit included in the constitution. France unveiled a 45 billion euro plan, while Italy's rescue plan – until now – amounts to 25 billion euro. Thus, actions have been left to national governments and European institutions have not been able to provide leadership in this emergency.

The lack of a coordinated action on fiscal policy in order to share the policies needed to confront the pandemic is a strong shortcoming of current EU action. Without a radical change, there could be strong asymmetries in the economic policy response and hard consequences for economy and well-being of those damaged countries whose policies will be constrained by a stricter path of adjustment of public expenditure.

Calls are mounting in Europe for much more decisive action. Facing the coronavirus pandemic, a strong European financial commitment and a change of its institutional set-up has been proposed by the former president of the European Commission Romano Prodi and Alberto Quadrio Curzio: "[T]he European Union has the tools to implement a project for the next decade which is able to mobilise, without any risks and with very limited costs, an increase in investment of at least 500 billion euros per year" (2020). The authors renewed the proposal to introduce Euro Union Bonds, based on the experience of the European Stability Mechanism (which may already issue European securities) and on the activities of the European Investment Bank. According to Quadrio Curzio, "[A] system with a central bank and a single currency must also have an adequate federal or confederal budget, between 10% and 20% of GDP, which can be financed with capital market issues" (2020a). Quadrio Curzio also proposed the issue of a 'Euro Rescue Bond' (ERB): "[W]ith appropriate guarantees, the ECB itself could purchase the ERBs as it has purchased government bonds of individual countries" (2020b). Such measures – a significant expansion of the European budget, the issuance of Eurobonds that the ECB can buy directly, a rethinking of the role of the European Stability Mechanism and the European Investment Bank to finance European public investment – are essential for turning the European Union into a political institution capable of facing the pandemic and its economic consequences, avoiding the current paralysis and the fragmentation of national responses.

On 25 March, an official letter by nine EU governments – Italy, France, Spain, Portugal, Slovenia, Greece, Ireland, Belgium and Luxembourg – demanded the creation of 'coronabonds', "a common debt instrument issued by a European institution to raise funds on the market on the same basis and to the benefits of all member states" (Dombey et al., 2020). The aim is to finance the necessary investment in health systems. Moreover, Italy's Prime Minister Giuseppe Conte demanded that Europe's response be "massive, cohesive, and timely" (ANSA, 2020).

If such proposal becomes official EU policy, a key step for changing European policies would be introduced. In a longer-term perspective, European fiscal policy should be based on a large common budget and a greater autonomy for national governments, starting with a 'golden rule' excluding public investment and all emergency-related expenditure from spending limits. European policies on expenditure, taxation and deficits must allow the development of the welfare state that is typical of Europe's model, favouring the convergence of member countries to high performance levels. In parallel, European policy must promote and finance efforts of all countries for restructuring their economy to prevent and adapt to climate change. Along these lines, Europe could become an international model, setting international standards on health, welfare and environmental issues, assuming a leadership in international organisations, identifying the most effective ways to face today's pandemic and tomorrow's climate emergency.

Unfortunately, there are no signals of change from the European Council, the European Commission and national governments; there is no political vision and capacity for action adequate to the severity of the current emergency. The coronavirus crisis may indeed become a crisis of European integration.

## What can we learn?

The economic consequences of the coronavirus pandemic are wide-ranging, affecting the way the world economy works. A number of lessons can be learned, starting with our views on health and the public good, on the possible changes in the relationships between health, economics and politics.

### Health is a global public good

The necessary starting point is a conception of health as a fundamental right. The Universal Declaration of Human Rights of the United Nations (UN) states that "[e]veryone has the right to a standard of living adequate for the health and well-being of himself and of his family, including food, clothing, housing and medical care and necessary social services" (1948). From an economic and public policy perspective, health is a global public good. It cannot be produced as a commodity and sold on the market to individual consumers, and is highly vulnerable to the lack of health – or, in fact, to the emergence of epidemics – in any part of the planet.

The importance of global public goods has been recognised in the late nineties in the context of the debate on globalisation:

> The United Nations Development Programme defines a global public good as 'a public good with benefits that are strongly universal in terms of countries (covering more than one group of countries), people (accruing to several, preferably all, population groups) and generations (extending to both current and future generations, or at least meeting the needs of current generations without foreclosing development options for future generations) (Kaul et al., 1999).

The specificity of health as a global public good has been at the centre of several studies (e.g. Smith et al., 2003), including its link to climate change (McMichael, 2013), and is now acknowledged even in World Bank policies for the prevention of pandemics (Stein and Sridhar, 2017). But the trajectory of the global economy has disregarded the need for global public goods. Neoliberal globalisation has prevailed, creating rules and institutions to protect the free movement of capitals and commodities only. New inter-governmental organisations were born – such as the World Trade Organization – and new global private powers ruled – the financial centres of Wall Street and the City, rating agencies, multinational corporations.

In those years, progressive European governments, trade unions, the International Labour Organization (ILO) and social movements had proposed to combine economic globalisation with new global protections for labour and social rights and the environment, as shown by the documents of the movements against the WTO summit in Seattle in 1999 and the Millennium Forum of civil society at the UN in 2000 (International Progress Organization, 2000).[3] They were ignored. No global rules and resources were introduced for welfare and health policies, for labour rights and environmental standards. These aspects were simply considered a 'cost' for the economy, left to fragmented national policies and put under pressure by privatisations and cuts in public resources.

The coronavirus pandemic has exposed the economic and social costs caused by the lack of adequate health and welfare systems in all countries and by the absence of global rules and coordination on the protection of health, from the markets of live animals in China to the ability to quickly identify and address an epidemic. The same holds for many environmental disasters – present and future ones – caused by climate change.

In order to address these global issues, we need to radically rewrite the rules of globalisation. Health, welfare, labour rights and the environment must be protected by international standards, which should be binding for the international movement of capitals and goods. The policy proposals put forward by the WHO, the ILO and the Climate Change conferences must acquire a new political priority and obtain the much-needed resources. The Sustainable Development Goals, endorsed by all UN member states, offer an additional framework in which to place these priorities (United Nations, 2015).

### The welfare state is an effective alternative to the market

Public health systems play a fundamental role in responding to the coronavirus pandemic. As Stiglitz (2020) argued, "when we face a crisis like an epidemic or a hurricane, we turn to government, because we know that such events demand collective action". Public health systems are based on a vision of health as a fundamental right that must be guaranteed by the government through the provision of universal public services designed to meet individual and social needs, outside the logic of the market. In Europe, this model has inspired the construction of the welfare state since the radical reforms introduced after World War II by the Labour Party in the UK. The welfare state, with its national varieties, remains at the core of the European 'social model'. Health, education, universities, pensions, social assistance and other key activities are provided and financed mainly by public action.

Three decades of neoliberal policies have seriously reduced the welfare state: privatisations and cuts in public budgets have forced public agencies to downsize their activities, sometimes losing universality, effectiveness and quality of services. Private companies have entered these activities, starting from the most profitable fields – pensions, healthcare and universities. Reduced funding, lack of turnover of personnel and pressure to make 'clients' pay for services have pushed welfare services to become more similar to market goods. The market system has been presented as the only way to effectively supply goods and services.

The pandemic has dramatically shown the price of such a neoliberal turn. Market globalisation creates health threats and is completely unable to respond to emergencies. Private health care is turning out to be largely irrelevant in facing the pandemic. The welfare state should not be considered a 'cost' for the private economic system. It is a parallel system that produces public goods and services and ensures the reproduction of society based on the rights and needs of citizens, rather than on the ability of customers to spend. The welfare state produces well-being and social quality, dimensions that the GDP, based on the market value of goods, cannot measure. The same arguments apply to environmental quality and the need for public intervention in this area; it is now urgently necessary to build accounting systems that seriously take into account social and environmental externalities produced by the economic systems.[4]

The obvious consequence of this analysis is that we should massively refinance – through a more progressive taxation of income and wealth and through deficit spending – a whole range of public activities: health, education, universities, research, pensions, social assistance and environmental protection.[5]

The welfare state could become the engine of a new model of development with high social quality and environmental sustainability. However, public policy should not be limited to the provision of welfare services. It must guide the development trajectories of the economy as a whole, ensuring consistency between business behaviour and the social, health and environmental goals mentioned above. In this regard, the debates on the return of industrial policy and on the 'European Green Deal' have

3 Several civil society documents are collected in Pianta (2001).

4 A novel measure of sustainable welfare for Italy is developed in Armiento (2018).

5 In Italy public spending has declined over time, slowly recovering in the last few years only. In terms of per capita healthcare expenditure the gap with the other major European countries has increased; the emphasis put on fiscal consolidation has left leave little room for social objectives. Italy is also characterised by a large divide on spending between Northern and Southern regions.

opened up a new space for public action at the national and European levels. There is growing consensus on expanding the role of the state and on the need for public action in the economy and society. An important example is provided by Mazzucato's proposals on the 'entrepreneurial state' and on the nationalisation of the pharmaceutical industry (2013; Mazzucato et al., 2020).

It would be a mistake to believe that, once the pandemic has passed, the economy could go back to 'normal'. We need to rethink production and consumption in light of health and environmental needs. There are other health crises that receive much less attention: occupational health and safety have been disregarded, and work-related accidents and deaths continue to be a dramatic issue. Pollution-related illnesses and deaths related to low environmental quality are a rising challenge in all countries. The 'deaths of despair' are a major social problem in the US and other countries, with a booming number of deaths, mainly of poor white men, related to suicide, alcohol, the use of opioids and drugs (Deaton and Case, 2020; Baldwin, 2020).

To confront such challenges, we have to move towards an economic system of greater quality, one which is able to cause less damage to the health of workers and citizens. Indeed, health and welfare could become a key engine of a novel development. In the current debate on the return of industrial policies, the report *What is to be produced?* proposes to identify three priority areas where public and private research and investment could be concentrated in order to develop a 'good' economy: environment and sustainability, knowledge and information and communication technologies, and health and welfare activities (Pianta et al., 2016). For the latter, as we argue in Pianta et al. (2019):

> Europe is an ageing continent with the best health systems in the world, rooted in their nature as a public service outside the market. Advances in care systems, instrumentation, biotechnologies, genetics and drug research have to be supported and regulated, considering their ethical and social consequences (as in the cases of GMOs, cloning, access to drugs in developing countries, etc.). Social innovation may spread in welfare services with a greater role of citizens, users and non-profit organisations, renewed public provision and new forms of self-organisation of communities. (279)

Such a policy can be built in Europe using existing institutions, policy tools and resources, pushing economic activities towards the protection of health and welfare and putting Europe on a sustainable path of long-term environmental transition on the basis of a green industrial policy. (Pianta et al., 2020; Lucchese and Pianta, 2020)

## The welfare state and public health reduce inequalities

Inequality is a major concern in this context. Since the 1980s, as a result of neoliberal policies, advanced countries have experienced sharp increases in income and wealth disparities. Because of its nature as a supplier of goods and services based on individual and social needs, the welfare state has been a key factor in reducing inequalities after the Second World War. As argued in our book *Explaining inequality* (Franzini and Pianta, 2015), the reduction of the policy space, the privatisation of public services and the extension of the market in areas previously protected by public action have introduced new mechanisms that generated economic and social disparities. In Europe a report from the European Commission recalled that "[i]n all countries with available data, significant differences in health exist between socioeconomic groups, in the sense that people with lower levels of education, occupation and/or income tend to have systematically higher morbidity and mortality rate" (2007). And, considering the economic effects of disparities in health conditions, the EU report calculated that the number of deaths that can be attributed to health inequalities in the European Union (EU25) as a whole is estimated to be 707,000 per year and the number of life years lost due to these death is about 11.4 million. Health inequalities also affect the average life expectancy at birth of men and women, decreasing it by 1.84 years. The total costs due to health inequalities – obtained from the combination of data relating to mortality and morbidity – is close to 980 billion euros, 9.38% of EU25 GDP in 2004. In other words, the loss of health due to socio-economic inequalities represents 15% of the costs of social security systems and 20% of the costs of health care systems in the European Union as a whole (Mackenbach, 2007).[6]

The relationship between inequalities and health has been analysed in several countries by considering different social and professional conditions (Wilkinson and Pickett, 2009; Costa et al., 2004), showing that mortality rates increase in proportion to economic and social hardship, lower incomes, education and social class. Thus, reducing economic inequalities would make it possible to reduce health disparities; at the same time, greater universal and egalitarian health protection would significantly reduce the costs of public health and welfare.

It is a paradox that the spreading of the pandemic today creates a condition of (almost) equality in the probability of contagion: in this situation, income levels matter relatively little and there is (almost) no way to 'buy' individual pro-

6 For a synthesis of the report, see https://www.epicentro.iss.it/politiche_sanitarie/diseg_economiche.

tection on the market. Equality in behaviours and health treatments becomes essential to fight the pandemic. But such equality can only be the result of universal public health, a fundamental outcome of the welfare state. As such, equality should be recognised as a key priority for the economic, social and health care policies of the post-coronavirus age.

The coronavirus pandemic is rapidly changing health conditions, daily life, social relationships and economic prospects around the world. It is important to learn the key lessons of its economic consequences and open up a debate on the possible actions that can set our societies on a more stable, healthy, egalitarian and sustainable trajectory of development.
